# Close negative correlation of local and circulating Dickkopf-1 and Sclerostin levels during human fracture healing

**DOI:** 10.1038/s41598-024-55756-5

**Published:** 2024-03-19

**Authors:** Julia Starlinger, Jonas Santol, Georg Kaiser, Kambiz Sarahrudi

**Affiliations:** 1 Department of Orthopedics and Trauma-Surgery, General Hospital Vienna, Medical University Vienna, Vienna, Austria; 2 Department of Surgery, HPB Center, Viennese Health Network, Clinic Favoriten, Sigmund Freud Private University, Vienna, Austria; 3 Division of Hepatobiliary and Pancreas Surgery, Department of Surgery, Mayo Clinic, Rochester, MN USA; 4 Department for Trauma Surgery, Wiener Neustadt Regional Hospital, Wiener Neustadt, Austria

**Keywords:** Dickkopf-1, DKK1, Fracture healing, Nonunion, Diagnostic markers, Bone

## Abstract

Wnt signaling is critically involved in fracture healing. Existing data predominantly relies on rodent models. Here, we explored local and circulating Dickkopf-1 (DKK1) levels in patients with respect to fracture healing and explore its association to sclerostin (SOST). 69 patients after surgical stabilization of long bone fractures of which six patients had impaired fracture healing were included in this study. Life-style and patient related factors with a known effect on DKK1 and SOST were recorded. DKK1 and SOST concentrations were measured using enzyme-linked immunosorbent assay (ELISA) at the fracture site and in circulation. DKK1 and SOST showed a close inverse correlation. In fracture hematoma and immediately after trauma DKK1 levels were significantly reduced while SOST levels were significantly increased, compared to healthy control. Postoperatively, DKK1 peaked at week 2 and SOST at week 8, again demonstrating a close negative correlation. Age and smoking status affected the balance of DKK1 and SOST, while type 2 diabetes and sex did not demonstrate a significant influence. Early postoperative elevation of SOST without compensatory DKK1 decrease was associated with fracture non-union in younger patients (< 50a). The close inverse correlation and very rapid dynamics of DKK1 and SOST locally as well as systemically suggest their critical involvement during human fracture healing. Importantly, as immediate compensatory feedback mechanism are apparent, we provide evidence that dual-blockade of DKK1 and SOST could be critical to allow for therapeutic efficiency of Wnt targeted therapies for fracture healing.

## Introduction

Fracture healing is a complex interplay of multiple processes, allowing for generation of new bone with structural and biomechanical integrity. In this context extensive experimental evidence has been generated on the relevance of these processes in rodent models^1^, however in human validation of these findings is largely missing. This is of particular relevance as non-osteogenic patient related factors such as diabetes, smoking and age critically affect human fracture healing and add a significant level of complexity to the pathophysiology on human fracture healing.

One of the experimentally well-defined processes involved in human fracture healing is the Wnt pathway^2^. While Wnt signaling plays a pivotal role in embryology as well as multiple regeneratory processes throughout the human body, is has been demonstrated to be critical in human fracture healing. It stimulates bone formation via osteoblasts and reduces osteoclastogenesis, thereby, reducing bone resorption^3,4^. It further supports the formation of bone and cartilage, particularly via its effect on osteogenic differentiation of mesenchymal stem cells^5^.

2 critical negative regulators of Wnt during bone healing have been identified, namely Dickkopf-1 (DKK1) and Sclerostin (SOST). Interaction with both have been documented to affect facture healing^6,7^, however, their effect seems to be significantly increased if both are targeted simultaneously^8^, suggesting a potential compensatory feedback mechanism of singe-target therapies. It is important to note that non-osteogenic, patient related factors such as age, sex, type 2 diabetes mellitus (T2DM) or smoking that are known risk factors for impaired fracture healing, have been demonstrated to critically affect circulating and intra-osseus DKK1 and SOST levels^9–14^.

While we previously reported on perioperative dynamics of SOST levels in human fracture healing, we now explored the interplay of DKK1 and SOST given accumulating evidence of their close intercorrelation. We assessed DKK1 and SOST levels in fracture hematoma and postoperative time course after surgical stabilization of long bone fractures, compare our findings in patients with functional vs. dysfunctional bone healing and assess the effect of non-osteogenic factors on their time course.

## Methods

Patient demographics are listed in Table 1. Serum of a consecutive series of 114 patients with meta-/diaphyseal fractures of long bone (humerus, femur, lower leg, and forearm) treated surgically at our institution between April 2006 and 2008, were collected. Exclusion criteria were: open fractures type III according to the Gustilo classification, previous bone operations, pre-existing bone diseases except for osteoporosis, renal/liver insufficiency, malignant tumors, long term steroid treatment, immunosuppression, and long term treatment with non-steroidal antiinflammatory drugs. Due to the strict selection criteria 39 patients with incomplete data were excluded from further investigation. Finally the data of 75 patients were analyzed. Patients were assigned to two groups according to their course of fracture healing. The first group contained 69 patients (male *n* = 31, female *n* = 38, mean age: 54.2 ± 20.5 years) with physiological fracture healing. Six patients were found to have impaired fracture healing and served as event cohort. Patients with impaired fracture healing all suffered from atrophic non-union. The diagnosis of bony consolidation or non-union was based on exercise-induced pain and conventional X-rays or computed tomography. Non-union was defined as the absence of complete consolidation at 6 months after surgery. In addition, 34 healthy volunteers (17 males, 17 females, mean age: 35.6 years) served as controls.

**Table 1:** Patient demographics.

	Fracture cohort (N = 114/100%)	Controls (N = 33/100%)
Parameter	Median (IQR)/N (%)	Median (IQR)/%
Age (years)	43.5 (32.75–58)	34.5 (29–46)
Sex		
Female	51.3%	55.9%
Male	48.7%	44.1%
Fracture location		
Femur	19.18%	
Lower leg	45,86%	
Femur + lower leg	7.02%	
Humerus	20.05%	
Forearm	7.89%	
Type of fixation		
Plate	33.33%	
IM	57.89%	
Ex. Fix	7.08%	
Screw	1.70%	
Soft tissue damage	14.91%	
Non Union (N = 7)	8.2%	
Non osteogenic risk factors		
Smoking	29.6%	
Diabetes	14.8%	
Frequent alcohol intake	7.4%	
DKK1 (pmol/L)		
BL		19.82 (16.06–26.77)
Week 1	20.68 (16.82–31.26)	
Week 2	27.22 (21.92–33.77)	
Week 4	21.31 (17.6–27.97)	
Week 6	21.21 (16.59–28.93)	
Week 8	21.59 (17–26.52)	
Week 12	18.18 (14.31–24.84)	
Week 24	19.58 (15.15–25.9)	
SOST (pmol/L)		
BL		8.19 (5.39–14.03)
Week 1	15.29 (10.7–22.36)	
Week 2	17.81 (12.65–26.24)	
Week 4	20.32 (15.4–27.6)	
Week 6	18.32 (13.97–27.69)	
Week 8	21.2 (15.98–31.79)	
Week 12	19.95 (14.2–32.01)	
Week 24	18.24 (13.7–27.65)	

IM, intramedullary fixation; Ex. fix., external fixator; Soft-tissue damage 1°, type I fracture due to Gutilo classification; BL, Baseline.

All patients were followed up for 12 months after the operation at our out-patients clinic. The follow-up examination was based on clinical and radiological examination at 1, 2, 4, 6, 8, 12, 24, and 48 weeks after trauma. In addition fresh fracture hematoma and peripheral venous blood was collected in 16 patients within 24 h after trauma.

Patients were further stratified according to non-osteogenic risk factors such as T2DM and smoking. Smoking status was defined based on self-reported cigarette consumption. 50 years of age served as the cut-off to differentiate between ‘young’ and ‘older’ patients. T2DM defined those patients under oral antidiabetic or insulin therapy.

All patients gave informed consent to be enrolled in this project and were 18–90 years old. The study was approved by the institutional ethics committee and was performed in accordance with the declaration of Helsinki.

### Blood samples

Samples were immediately centrifuged at 1000 × *g* for 10 min at 4 °C and the resulting serum was stored at − 80 °C until analysis. Fracture hematoma was obtained at surgery. Hematoma was removed manually before any manipulation or irrigation, avoiding contamination by blood in the operating field, and placed in sterile containers. These specimens were centrifuged immediately 1000 × *g* for 10 min at 4 °C and the resulting supernatant was stored at − 80 °C until assayed.

### Measurement of DKK1 and SOST

DKK1 and SOST concentrations were measured by a commercially available enzyme-linked immuno sorbent assay (ELISA). All analytical steps were performed according to the manufacturer's recommended protocol. Concentrations are presented as mean of duplicate measurements.

### Statistical analysis

Comparisons between independent groups of continuous variables were performed by non-parametric Mann–Whitney U-test or Fisher's Exact Test where applicable. For comparisons of serum with fracture hematoma and of the serum values in patients with impaired healing before and after re-operation non-parametric Wilcoxon-test for paired samples was used. Statistical analyses were performed using SPSS for Windows 23.0 and GraphPad. Data are presented as means ± SEM (standard error of the mean). The statistical significance level was set at *P* < 0.05.

### Ethics approval

This study was approved by the Ethic Committee of the Medical University of Vienna Nr.810/2010.

## Results

### DKK1 decreases immediately after long bone fracture, shows exceedingly low concentrations in the fracture hematoma and peaks 2 weeks after surgical intervention

**Figure 1:**
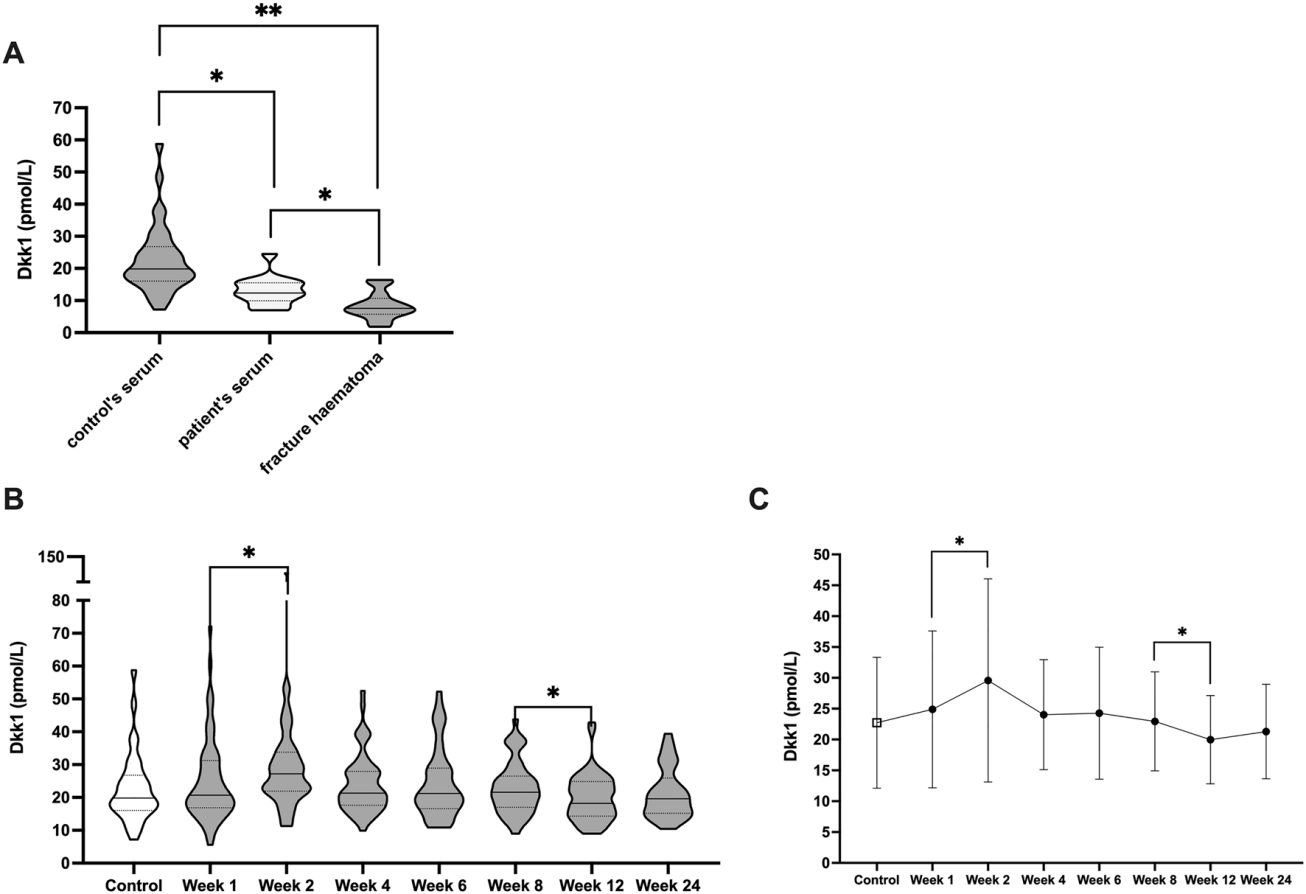
DKK1 dynamics during human fracture healing. (**A**) illustrates and compares circularing DKK1 levels in healthy controls, patients immediately after traumatic long bone fracture as well as the aspirated fracture hematoma. (**B**) shows DKK1 time course during fracture healing as well as compares it to healthy controls (respective medians and standard deviations are illustrated as line diagram for better time course evaluation in (**C**). *P < 0.05, **P < 0.005.

Given the critical relevance of DKK1 and SOST in fracture healing, we initially assess circulating DKK1 levels in controls to establish baseline levels in healthy individuals (Median concentration: 19.8 pmol/l, N = 34). Importantly, when comparing circulating DKK1 levels of these controls to patients immediately upon diagnosis of long-bone fracture, we observed that DKK1 levels were significantly lower in these patients (Median concentration 12.35 pmol/l, *P* = 0.006, N = 69.). Of note, in the fracture hematoma (N = 16), we found even lower levels of DKK1 than in patients and controls serum (Median concentration 7.57 pmol/l, Controls: *P* < 0.001, Patients: *P* = 0.022 Fig. 1A).

Given the significant immediate differences we observed of circulating DKK1 as well as in the fracture hematoma, we further aimed to assess DKK1 concentrations during the subsequent weeks of fracture healing. Importantly, we observed that already 1 week after surgical management of the fracture, DKK1 levels returned to levels comparable to healthy control individuals (Median concentration postoperative week 1 20.68 pmol/L, Fig. 1B,C). However, circulating DKK1 levels further increased till postoperative week 2 and reached significantly higher levels than in healthy controls (*P* = 0.014, Fig. 1B,C). After this peak, circulating DKK1 levels continuously decreased during the further fracture healing process, leveling around healthy control concentrations (Fig. 1B,C).

### DKK1 inversely correlate with SOST levels and show distinct dynamics in patients with dysfunctional fracture healing

**Figure 2:**
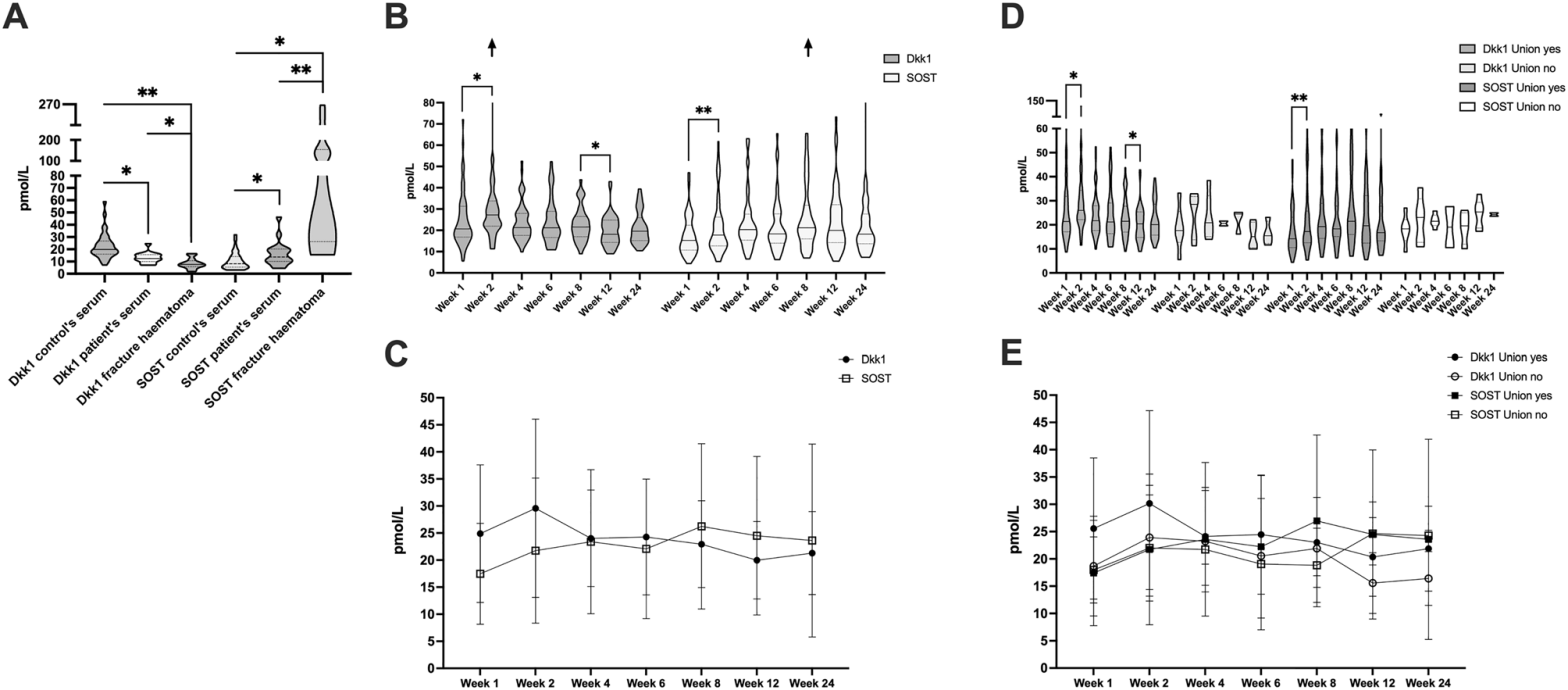
DKK1 and SOST are closely negatively correlated. (**A)** compares DKK1 and SOST levels in healthy controls, patients immediately after traumatic long bone fracture as well as the aspirated fracture hematoma. (**B**) compares time course of DKK1 and SOST during fracture healing and arrows indicate their peak (respective medians and standard deviations are illustrated as line diagram for better visualization of time course in (**C**)—inverse correlation**—P** = 0.009**)**. (**D**) compares DKK1 and SOST levels time course in patients with and without dysfunctional bone healing (respective medians and standard deviations are illustrated as line diagram for better visualization of time course in **E**). *P < 0.05, **P < 0.005.

Given the well documented close correlation of DKK1 and SOST we further aimed to compare their levels immediately after trauma in the facture hematoma. Already at this early stage we confirmed an inverse correlation of DKK1 and SOST. As mentioned above, while DKK1 levels strikingly decreased in facture hematoma, we found SOST levels significantly elevated at this early stage of fracture healing (Fig. 2A). When we further compared their postoperative time course, we observed that also during this prolonged time frame of fracture healing, SOST levels decreased when DKK1 levels were elevated and vice versa (Fig. 2B,C, inverse correlation: *P* = 0.009). In line with these results, we found that circulating DKK1 peaked relatively rapidly during fracture healing at 2 weeks, while SOST levels only peaked in week 8, after DKK1 levels were declining again on (Fig. 2B,C). We further aimed to compare the postoperative circulating DKK1 and SOST time course in patients with impaired fracture healing. While not statistically significant, we found continuously lower DKK1 levels in patient with non-union (Fig. 2D,E). Otherwise, the time-course mirrored patients with functional fracture healing. Importantly, while not significant, patients with non-union appeared to level at a lower DKK1 concentration than healthy controls at 24 weeks of fracture healing (Median concentration non-union patients at 24 weeks 15.45 pmol/L vs. Median concentration healthy controls 20.09 pmol/L). Results on SOST have been partially previously been reported^15^, but are illustrated to allow for direct comparison.

### Non-osteogenic risk factors critically affect DKK1 levels

**Figure 3:**
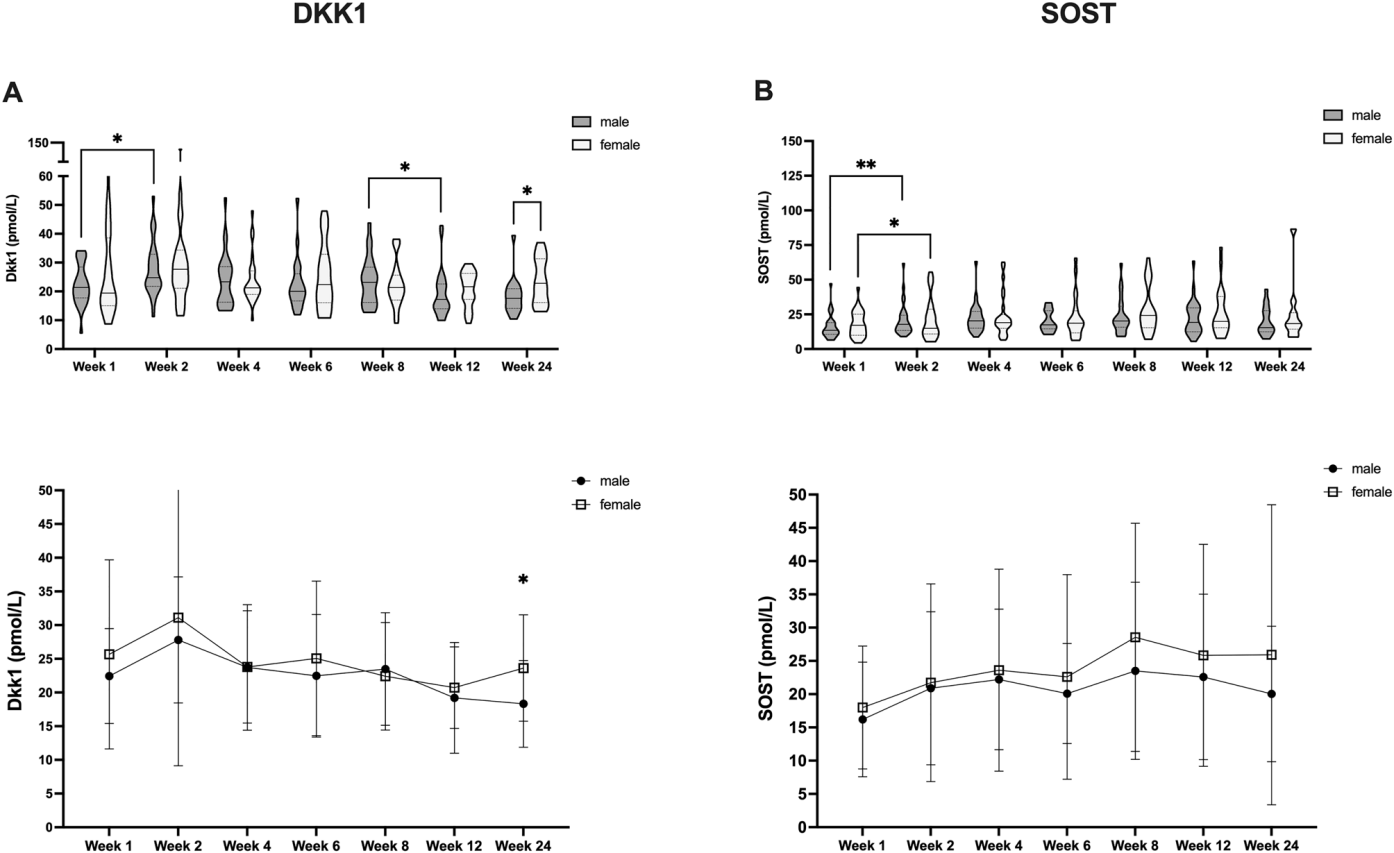
Effect of patient sex on circulating DKK1 and SOST levels during fracture healing. DKK1 and SOST levels during the time course of fracture healing are compared regards to sex (DKK1 (**A**) top: violin plots, bottom: line diagram with medians and standard deviations; SOST (**B**) top: violin plots, bottom: line diagram with medians and standard deviations). *P < 0.05, **P < 0.005.

**Figure 4:**
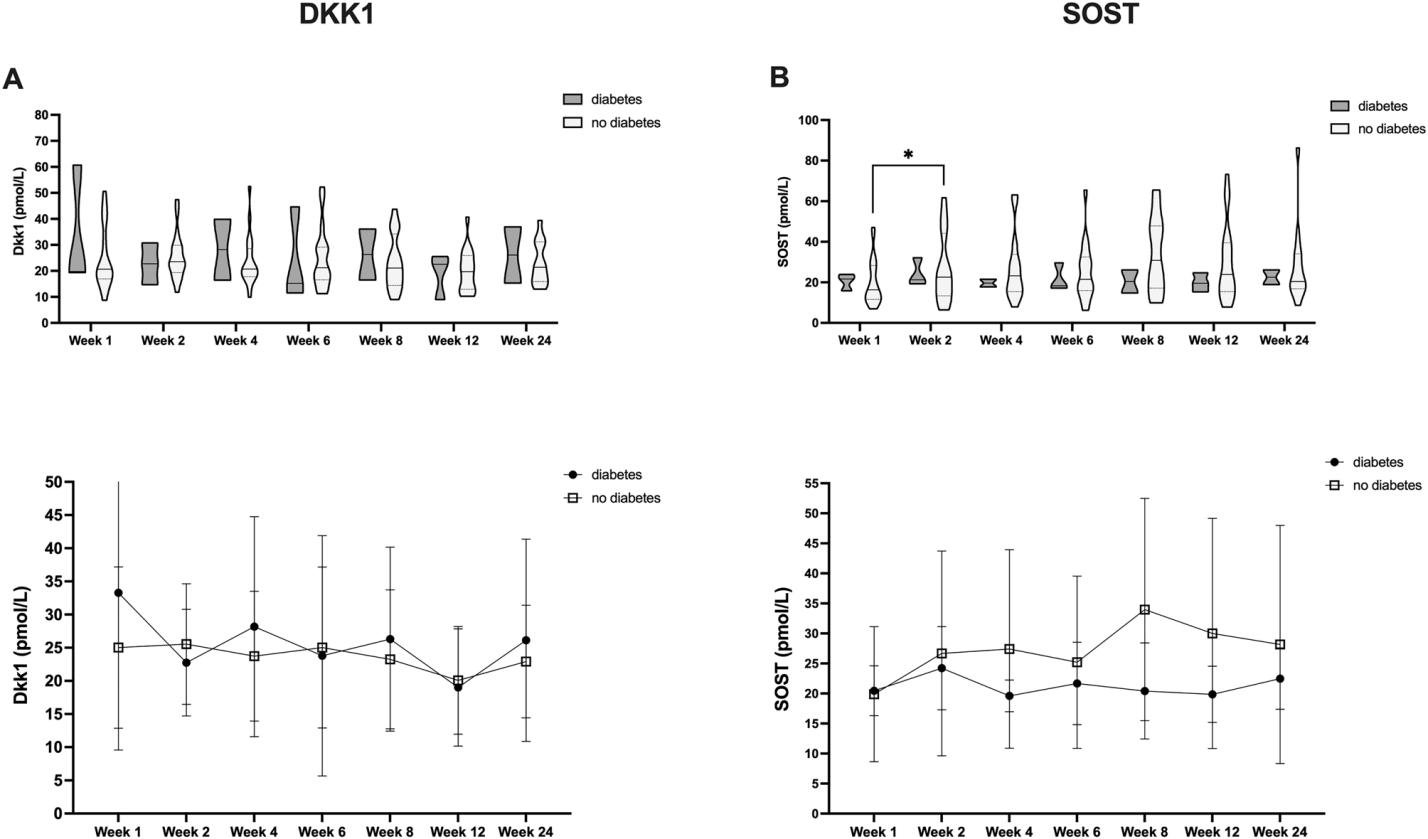
Effect of T2DM on circulating DKK1 and SOST levels during fracture healing. DKK1 (**A**) top: violin plots, bottom: line diagram with medians and standard deviations; SOST (**B**) top: violin plots, bottom: line diagram with medians and standard deviations). *P < 0.05, **P < 0.005.

**Figure 5:**
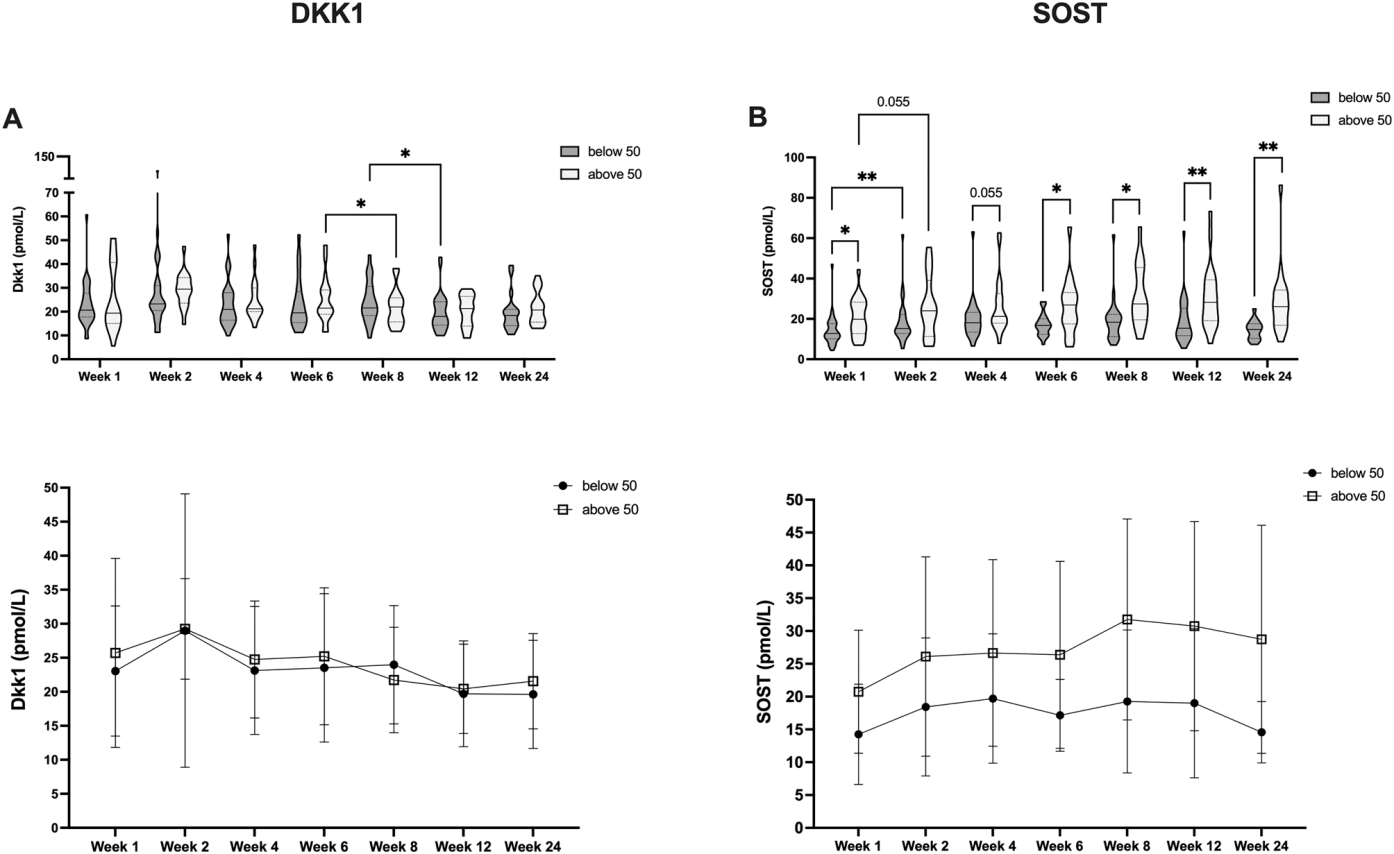
Effect of age on circulating DKK1 and SOST levels during fracture healing. DKK1 (**A**) top: violin plots, bottom: line diagram with medians and standard deviations; SOST (**B**) top: violin plots, bottom: line diagram with medians and standard deviations).

**Figure 6:**
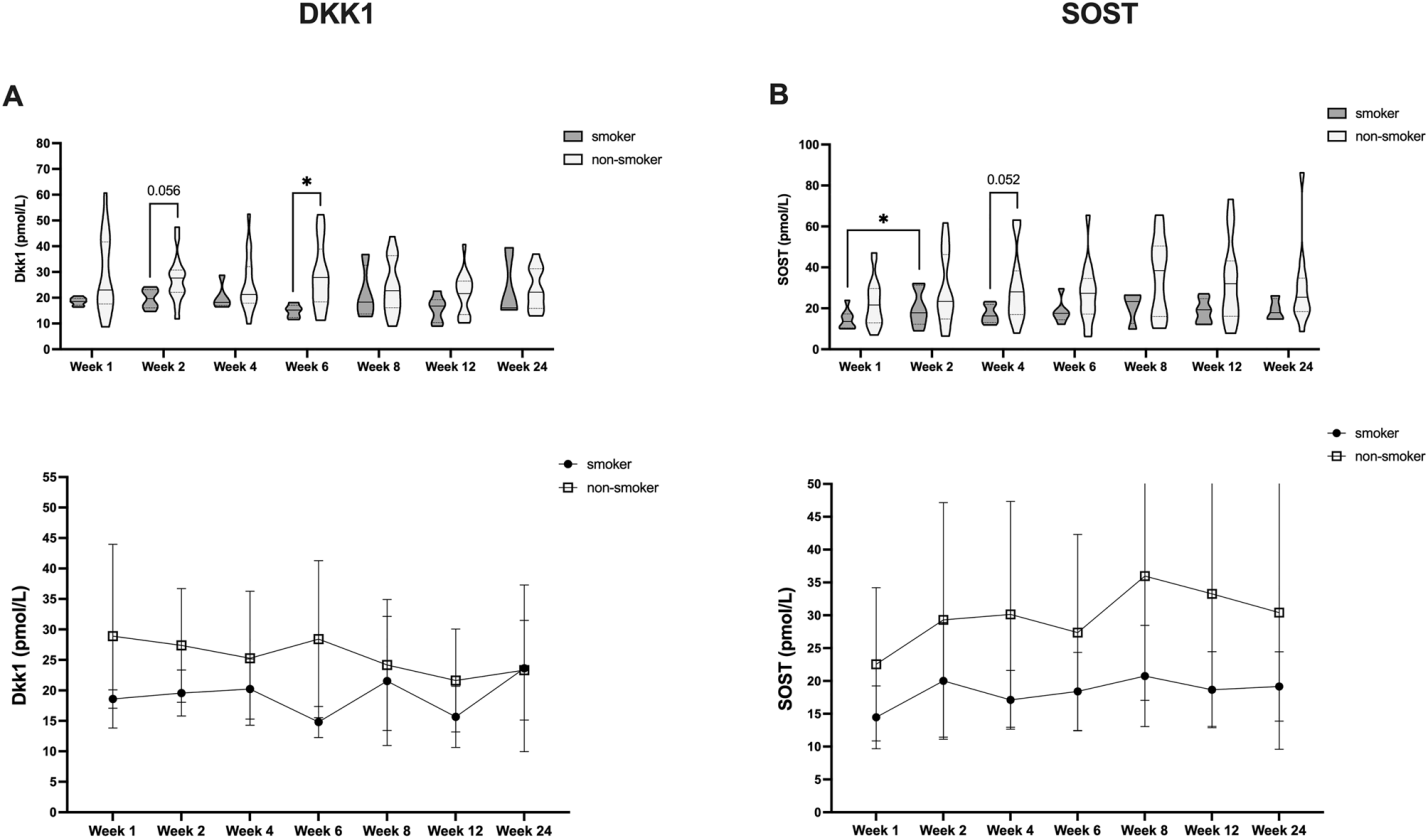
Effect of smoking on circulating DKK1 and SOST levels during fracture healing. DKK1 (**A**): op: violin plots, bottom: line diagram with medians and standard deviations; SOST (**B**)**:** op: violin plots, bottom: line diagram with medians and standard deviations). *P < 0.05, **P < 0.005.

Given extensive preexisting evidence that non-osteogenic factors could affect fracture healing via effects on DKK1 and SOST, we further assessed if non-osteogenic parameters were affecting protein dynamics during human fracture healing. Accordingly, we sought to subclassify patients further with respect to these characteristics (Fig. 3). Overall, we did not observe striking differences in DKK1 and SOST time courses during fracture healing according to sex (Fig. 3A,B) and T2DM (Fig. 4A,B). However, we noted older patients, had significantly higher circulating SOST levels after surgery, without a compensatory DKK1 reduction (> / ≤ 50a, Fig. 5A,B). Further, we found that smokers had consistently lower circulating DKK1 and SOST levels (Fig. 6A,B).

### Young patients with non-union show higher SOST and DKK1 levels during the early phase of fracture healing

**Figure 7:**
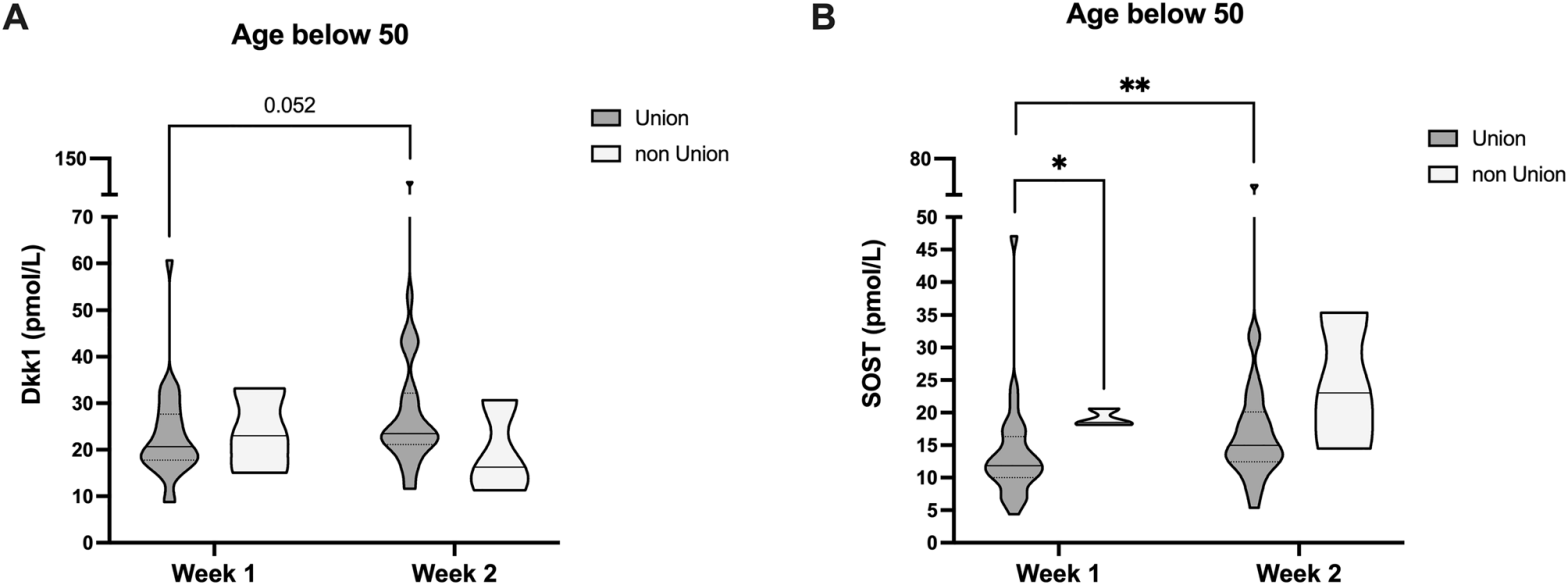
Increased SOST levels, without compensatory DKK1 decrease, during the early phase of bone regeneration are associated with dysfunctional fracture healing in young patients. DKK1 and SOST levels in patients < 50a of age, stratified according to the further development of non-union. *P < 0.05, **P < 0.005.

As we observed the most striking differences in circulating DKK1 and SOST levels in age, we further aimed to assess if patients with dysfunctional bone healing would differ in terms of early SOST or DKK1 levels in these subgroups. And indeed, we observed that younger patients (below 50a) with dysfunctional fracture healing did show higher SOST and DKK1 levels within the early phase of bone healing (first 2 weeks, SOST: *P* = 23.03 pmol/L, DKK1: *P* = 16.28 pmol/L Fig. 7).

## Discussion

Impaired fracture healing represents a major complication after surgical intervention for long bone fractures and dramatically affects patient quality of life. SOST and DKK1 are increasingly recognized as critical regulators of bone metabolism and healing^16^ However, while experimental research has documented the relevance of DKK1 and SOST in Wnt inhibition during fracture healing in rodents, the relevance in humans, with different patient related morbidities and life style factors, is largely missing. Within this analysis, we were now able to document that DKK1 levels rapidly decrease after long bone fractures and are exceedingly low in the fracture hematoma. However, this reduction was only temporary and recovery to normal (compared to healthy controls) levels within only one week. Presumably, as a negative feedback mechanism, we found that DKK1 levels further increased, to significantly elevated levels on postoperative week 2 where day peaked, suggesting a possible role in regulating fracture healing after an initial response to the traumatic insult. Importantly, we found that local and circulating DKK1 levels to very closely inversely correlate with SOST concentrations, indicating that the previously suggested compensatory feedback loop, might indeed also be relevant in human fracture healing. While we did not observe significant differences in patients with dysfunctional fracture healing, these patients appeared to have consistently lower levels of DKK1 during the process of fracture healing. Ultimately, we only found age and smoking to significantly affect circulating SOST and DKK1 levels during fracture healing and provide exploratory evidence that younger patients with impaired fracture healing show an increased levels of SOST during the early period of fracture healing. To our knowledge, this is the first report exploring the time course of DKK1 and its correlation to SOST during a prolonged period of in human fracture healing and providing indispensable information for continues aspirations to utilize single or dual targeted therapies against DKK1 and SOST.

In rodents SOST and DKK1 levels increased in maturing osteocytes of the external callus after day 7, with DKK-1 induction also observed on day 3 in the periosteal region adjacent to the fracture line, returning towards baseline levels as bony bridging was established at week 5^8^. Within our analyses we were able to demonstrate that also after human long bone fractures, DKK1 and SOST are significantly affected, suggesting a critical role also during human fracture healing. Of note, their time course appeared very distinct and our analyses demonstrated a remarkable negative correlation of DKK1 and SOST levels. This was most appreciable in the fracture hematoma, where SOST levels were exceedingly high while DKK1 levels were significantly reduced. Similar to the time course during fracture healing, it appeared that SOST could only increase if DKK1 levels were returned to normal levels. In this context circulating DKK1 peaked rapidly during fracture healing (2 weeks) while SOST peaked relatively delayed at week 8. This presumably compensatory feedback counter-regulation of SOST and DKK1 has previously been described in experimental models^17^, but in human validation of this correlation has been limited. In a setting different from fracture healing, namely in patients with sclerostosis, characterized by deficient sclerostin synthesis and augmented bone formation, increased circulating DKK1 levels have been reported, which goes in line with our observation^18^. It is important to note that, while DKK1 as well as SOST negatively regulate Wnt signaling and clearly have synergistic effects, they do appear to exert very distinct effects on the low density lipoprotein receptor-related proteins 5 and 6^19^. Accordingly, it has been suggested that sclerostin has evolved as a more refined regulator of Wnt signaling, whereas DKK1, given its more ancient phylogeny and pan-Wnt inhibitory activity, may function more widely as a brake on Wnt signaling^19^. In line with this hypothesis, DKK1 might demonstrate its first peak rather early do avoid overwhelming bone regeneration, while SOST might be more critical during later stages of fracture healing. In this context it is important to note that serum SOST levels have been shown to predict for bone formation, while DKK1 levels were associated with bone resorption in patients with T2DM ^20^, which would go in line with this hypothesis.

In long bone fracture models, fracture healing comprises of 2 major components: (1) endochondral and (2) intramembranous ossification. Cartilaginous callus is replaced by bone during endochondral ossification, whereas intramembranous ossification is a result of direct bone apposition. As both aspects are affected by Wnt signaling, targeting DKK1 or/and SOST have been proposed as therapeutic targets. In this context, a SOST-Antibody has been shown to accelerate intramembranous ossification^16,21,22^. Further, given that large gap-defects also primarily heal by direct bone apposition, increased bridging and mineralized bone mass has been reported for SOST-Antibody treatment in critical-sized defects in the rat femur^16^. Similar results have been reported for specific DKK1-blockage^6,23^. However, as mono-targeted therapies have been shown to suffer from compensatory feedback loops^8,17^, dual inhibition of DKK1 and SOST has been tested and demonstrated even superior fracture healing in rodents and non-human primates than with mono-targeted therapies^8^. As we observed a close inverse correlation of DKK1 and SOST in human fracture hematoma as well as during the fracture healing time course, our data would suggest that also in humans dual inhibition of DKK1 and SOST would most likely be critical to improve fracture healing. This might ultimately, in part, explain why clinical trial investigating the effects of mono-targeted SOST inhibition have ultimately failed to improve fracture healing^24,25^.

This is the first study to assess circulating DKK1 and SOST levels with their respective time course after long bone fracture healing following surgical stabilization. Importantly, we also explored and compared the effects of non-osteogenic, patient related factors in this context. We found that the most significant factors affecting SOST and DKK1 levels during human fracture healing were age and smoking. Indeed, older patients did demonstrate consistently elevated SOST levels during the entire fracture healing period, suggesting a critical relevance of age in Wnt associated bone healing, which is in line with existing literature^26^. Importantly, we found that in younger patients increased levels of SOST during the early period of fracture healing was associated with dysfunctional fracture healing. While these results are exploratory in nature, they demonstrate that there might be a subgroup of patients that might particularly benefit from Wnt targeting therapies to avoid non-union of long bone fractures. Of interest, while we very consistently observed an inverse correlation of DKK1 and SOST during the fracture healing time course, in this non-union subgroup of young patients, both appeared elevated, suggesting a disruption of the inverse balance of DKK1 and SOST in these patients.

A tight regulation of osteoclast function is of critical importance during secondary fracture healing^27^. Our results further strengthen their crucial importance and their balanced regulation during this process for several weeks in humans. While osteoclast gene expression can be affected by several factors and varies during differentiation^28^, our results would suggest that the functional state of osteoclasts, determine their overall effect during long bone fracture healing in humans.

T2DM and sex have also been documented to significantly affect respective Wnt signaling pathways and circulating DKK1 and SOST levels^20,26^. While limited in overall power, we did not find any striking differences in SOST and DKK1 time course in our patients. Importantly, we noticed that the effects of age and smoking status appears to outweigh these other non-osteogenic factors in terms of their effects on circulating DKK1 and SOST levels in human long bone fracture healing.

With increasing age, both circulating DKK1 and SOST levels increase^29,30^. It is important to note that our healthy control individuals were significantly younger than our fracture cohort. Of note, while we indeed observed increased SOST levels in this cohort, DKK1 levels were decreased in the fracture cohort, despite their higher age. This could suggest a predisposition of these patients for long bone fractures, but could also be caused by their underlying comorbidities or smoking status. This observation will need further exploration in future analyses.

Our analyses have several intrinsic limitations that should be taken into account when interpreting our results. Despite strict inclusion and exclusion criteria, a given heterogeneity within the patient group represents a potential bias. In particular, not only do fracture morphology and location vary to some extent between patients, but the corresponding type of fracture healing may also differ with respect to the operative procedure, such as nailing or plating. While this is certainly to some extent the nature of translational research, this could in part have affected our results. However, facture location and subsequent treatment, was distributed equally within our groups. We believe it is worth noting that, as we still observed significant differences in regards to nonosteogenic factors, reported associations seem to overcome some heterogeneity and biological variability within our cohort. Further, while we present a very detailed time course of circulating DKK1 and SOST during human fracture healing and within the fracture hematoma and do believe that we provide very valuable information on the time course of these proteins, we are certainly limited in overall statistical power given the relatively small samples size of our analysis. This limited power also led us to omit correction for multiple testing. Accordingly, future confirmatory analyses will be necessary. It also needs to be noted that within this study design no bone tissue was collected during surgery. This limits us in several important confirmatory assessments, such as if the fracture hematoma SOST and DKK1 levels actually correlate with the bone contents of these factors using immunohistochemical stainings, we were also unable to assess the bone mineral density, bone microarchitecture, and bone strength, which needs to be taken into account when interpreting our results.

In conclusion, with this analysis we can provide preliminary results on the dynamics of DKK1 and SOST after human traumatic long bone fracture. Most significantly, they showed a very distinct and closely inversely correlated dynamic which was in part affected by non-osteogenic/patient related factors such as smoking and age. In younger patients increased postoperative SOST levels (without compensatory DKK1 reduction) were observed in patients with dysfunctional fracture healing. Our results provide critical translational insight into the possible time dependent involvement of DKK1 and SOST in human fracture healing and their association with patient related factors and their outcome but most importantly suggest that very rapid compensatory feedback loops between DKK1 and SOST are active and might in part explain the lack of therapeutic efficiency of mono-targeted Wnt directed therapies.

## Data Availability

The datasets generated and/or analysed during this study are not publicly available due to sensitive patient data but are available from the corresponding author on reasonable request.

## References

[1] Einhorn TA, Gerstenfeld LC (2015). Fracture healing: Mechanisms and interventions. Nat. Rev. Rheumatol..

[2] Houschyar KS, Tapking C, Borrelli MR, Popp D, Duscher D, Maan ZN (2018). Wnt pathway in bone repair and regeneration—What do we know so far. Front. Cell Dev. Biol..

[3] Krishnan V, Bryant HU, Macdougald OA (2006). Regulation of bone mass by Wnt signaling. J. Clin. Investig..

[4] McDonald MM, Morse A, Schindeler A, Mikulec K, Peacock L, Cheng T (2018). Homozygous Dkk1 knockout mice exhibit high bone mass phenotype due to increased bone formation. Calcif. Tissue Int..

[5] Tang N, Song WX, Luo J, Luo X, Chen J, Sharff KA (2009). BMP-9-induced osteogenic differentiation of mesenchymal progenitors requires functional canonical Wnt/beta-catenin signalling. J. Cell Mol. Med..

[6] Jin H, Wang B, Li J, Xie W, Mao Q, Li S (2015). Anti-DKK1 antibody promotes bone fracture healing through activation of β-catenin signaling. Bone.

[7] Mihara A, Yukata K, Seki T, Iwanaga R, Nishida N, Fujii K (2021). Effects of sclerostin antibody on bone healing. World J. Orthop..

[8] Florio M, Gunasekaran K, Stolina M, Li X, Liu L, Tipton B (2016). A bispecific antibody targeting sclerostin and DKK-1 promotes bone mass accrual and fracture repair. Nat. Commun..

[9] Tsentidis C, Gourgiotis D, Kossiva L, Marmarinos A, Doulgeraki A, Karavanaki K (2017). Increased levels of Dickkopf-1 are indicative of Wnt/β-catenin downregulation and lower osteoblast signaling in children and adolescents with type 1 diabetes mellitus, contributing to lower bone mineral density. Osteoporos Int..

[10] Faienza MF, Ventura A, Delvecchio M, Fusillo A, Piacente L, Aceto G (2017). High Sclerostin and Dickkopf-1 (DKK-1) serum levels in children and adolescents with type 1 diabetes mellitus. J. Clin. Endocrinol. Metab..

[11] Hie M, Iitsuka N, Otsuka T, Tsukamoto I (2011). Insulin-dependent diabetes mellitus decreases osteoblastogenesis associated with the inhibition of Wnt signaling through increased expression of Sost and Dkk1 and inhibition of Akt activation. Int. J. Mol. Med..

[12] Hildebrandt N, Colditz J, Dutra C, Goes P, Salbach-Hirsch J, Thiele S (2021). Role of osteogenic Dickkopf-1 in bone remodeling and bone healing in mice with type I diabetes mellitus. Sci. Rep..

[13] Pacicca DM, Brown T, Watkins D, Kover K, Yan Y, Prideaux M (2019). Elevated glucose acts directly on osteocytes to increase sclerostin expression in diabetes. Sci. Rep..

[14] Jorde R, Stunes AK, Kubiak J, Grimnes G, Thorsby PM, Syversen U (2019). Smoking and other determinants of bone turnover. PloS One.

[15] Sarahrudi K, Thomas A, Albrecht C, Aharinejad S (2012). Strongly enhanced levels of sclerostin during human fracture healing. J. Orthop. Res..

[16] Ke HZ, Richards WG, Li X, Ominsky MS (2012). Sclerostin and Dickkopf-1 as therapeutic targets in bone diseases. Endocr. Rev..

[17] Witcher PC, Miner SE, Horan DJ, Bullock WA, Lim KE, Kang KS (2018). Sclerostin neutralization unleashes the osteoanabolic effects of Dkk1 inhibition. JCI Insight.

[18] van Lierop AH, Moester MJ, Hamdy NA, Papapoulos SE (2014). Serum Dickkopf 1 levels in sclerostin deficiency. J. Clin. Endocrinol. Metab..

[19] Ren Q, Chen J, Liu Y (2021). LRP5 and LRP6 in Wnt signaling: Similarity and divergence. Front. Cell Dev. Biol..

[20] Wang N, Xue P, Wu X, Ma J, Wang Y, Li Y (2018). Role of sclerostin and dkk1 in bone remodeling in type 2 diabetic patients. Endocr. Res..

[21] McDonald MM, Morse A, Mikulec K, Peacock L, Yu N, Baldock PA (2012). Inhibition of sclerostin by systemic treatment with sclerostin antibody enhances healing of proximal tibial defects in ovariectomized rats. J. Orthop. Res..

[22] Ominsky MS, Li C, Li X, Tan HL, Lee E, Barrero M (2011). Inhibition of sclerostin by monoclonal antibody enhances bone healing and improves bone density and strength of nonfractured bones. J. Bone Miner. Res..

[23] Li X, Grisanti M, Fan W, Asuncion FJ, Tan HL, Dwyer D (2011). Dickkopf-1 regulates bone formation in young growing rodents and upon traumatic injury. J. Bone Miner. Res..

[24] Bhandari M, Schemitsch EH, Karachalios T, Sancheti P, Poolman RW, Caminis J (2020). Romosozumab in skeletally mature adults with a fresh unilateral tibial diaphyseal fracture: A randomized phase-2 study. J. Bone Joint Surg. Am..

[25] Schemitsch EH, Miclau T, Karachalios T, Nowak LL, Sancheti P, Poolman RW (2020). A randomized, placebo-controlled study of romosozumab for the treatment of hip fractures. J. Bone Joint Surg. Am..

[26] Mödder UI, Hoey KA, Amin S, McCready LK, Achenbach SJ, Riggs BL (2011). Relation of age, gender, and bone mass to circulating sclerostin levels in women and men. J. Bone Miner. Res..

[27] ElHawary H, Baradaran A, Abi-Rafeh J, Vorstenbosch J, Xu L, Efanov JI (2021). Bone healing and inflammation: Principles of fracture and repair. Semin. Plast. Surg..

[28] Toor SM, Wani S, Albagha OME (2021). Comprehensive transcriptomic profiling of murine osteoclast differentiation reveals novel differentially expressed genes and LncRNAs. Front Genet..

[29] Dovjak P, Dorfer S, Föger-Samwald U, Kudlacek S, Marculescu R, Pietschmann P (2014). Serum levels of sclerostin and Dickkopf-1: Effects of age, gender and fracture status. Gerontology.

[30] Hay E, Bouaziz W, Funck-Brentano T, Cohen-Solal M (2016). Sclerostin and bone aging: A mini-review. Gerontology..

